# Comparison of OCT Structural Parameters and OCTA‐Measured Retinal Vessel Density and Peripapillary Choroidal Microvasculature in Glaucoma and Optic Neuritis From Multiple Sclerosis or Neuromyelitis Optica

**DOI:** 10.1155/joph/6354194

**Published:** 2026-07-16

**Authors:** Glauco Batista Almeida, João Américo Domingos, Marcelo Hatanaka, Leandro Cabral Zacharias, Leonardo Provetti Cunha, Luiz Guilherme Marchesi Mello, Mário Luiz Ribeiro Monteiro

**Affiliations:** ^1^ Division of Ophthalmology and the Laboratory of Investigation in Ophthalmology (LIM 33), University of São Paulo, São Paulo, Brazil, usp.br; ^2^ Department of Ophthalmology, Federal University of Mato Grosso do Sul, Campo Grande, Mato Grosso do Sul, Brazil, ufms.br; ^3^ Department of Neurology, Federal University of Mato Grosso do Sul, Campo Grande, Mato Grosso do Sul, Brazil, ufms.br; ^4^ Department of Ophthalmology, Federal University of Juiz de Fora Medical School, Juiz de Fora, Minas Gerais, Brazil; ^5^ Ophthalmology Unit, Hospital Universitário Cassiano Antônio Moraes, Federal University of Espírito Santo, Vitoria, Espírito Santo, Brazil, ufes.br

**Keywords:** choroidal microvasculature, glaucoma, macular ganglion cell complex, multiple sclerosis, neuromyelitis optica, optical coherence tomography angiography, standard automated perimetry, vessel density

## Abstract

**Aims:**

To compare peripapillary retinal nerve fiber layer (pRNFL), choroidal microvascular dropout (CMvD) and vessel density (pVD), as well as macular ganglion cell complex (GCC) and vessel density (mVD) in eyes with glaucoma and optic neuritis (ON) due to multiple sclerosis (MS‐ON) or neuromyelitis optica spectrum disorder (NMOSD‐ON) using OCT and OCT angiography (OCTA), and to assess its correlations with visual acuity and visual field (VF) parameters.

**Methods:**

Thirty‐one eyes (16 patients) with glaucomatous optic neuropathy (GON), 26 eyes (16 patients) with MS‐ON, 19 eyes (12 patients) with NMOSD‐ON, and 53 healthy eyes (27 subjects) underwent 24‐2 VF, OCT, and OCTA. The pRNFL thickness, pVD, and CMvD, as well as macular GCC thickness and mVD, were analyzed and compared using generalized estimating equations. Area under the receiver operating characteristic curves was calculated.

**Results:**

All disease groups showed significantly reduced structural parameters compared to controls. MS‐ON and NMOSD‐ON did not differ and demonstrated greater GCC thinning than GON despite similar pRNFL thickness (except nasally). mVD did not significantly differ among disease groups. In contrast, superficial and deep pVD distinguished the disease groups from controls and were significantly lower in nasal and temporal sectors in MS‐ON and particularly in NMOSD‐ON compared to GON. Although CMvD occurred in all groups, it was significantly more frequent and extensive in GON. Significant correlations were found between OCTA and OCT and visual function parameters in MS‐ON and NMOSD‐ON but not in GON eyes.

**Conclusions:**

OCT and OCTA parameters seem to differentiate GON, MS‐ON, and NMOSD‐ON from controls, but substantial overlap exists among the diseases. The presence of CMvD, particularly when extensive, was more commonly observed in GON, although its presence in other groups limits its diagnostic specificity.

**Trial Registration:** Brazilian Clinical Trials Registry: 76722517.2.0000.0068

## 1. Introduction

Optical coherence tomography (OCT) is a widely used technique that enables high‐resolution visualization and measurement of the retina, choroid, and optic nerve head (ONH) structures [1]. It has become an essential tool for the evaluation and monitoring of patients with various conditions, including glaucoma, optic neuritis (ON), compressive optic neuropathies, and neurodegenerative disorders [2–4]. Over the past years, advances in OCT technology have significantly enhanced the understanding of structural changes in these diseases, allowing precise quantification of segmented retinal layers, the peripapillary retinal nerve fiber layer (pRNFL), and other ONH parameters [2, 5, 6].

Glaucoma and ON are major anterior visual pathway disorders associated with visual loss and structural damage to the retina and optic nerve [7, 8]. Glaucoma is the leading cause of irreversible blindness worldwide, with primary open‐angle glaucoma being the most common subtype [8]. Despite its high prevalence, the exact pathophysiology of glaucoma remains incompletely understood [9]. ON, in contrast, is an inflammatory optic neuropathy and a frequent manifestation of autoimmune central nervous system diseases, such as multiple sclerosis (MS) and neuromyelitis optica spectrum disorder (NMOSD) [7, 9]. Despite their different pathophysiology, both glaucoma and ON lead to significant retinal and optic nerve changes [10, 11].

Distinguishing glaucomatous optic neuropathy (GON) from inflammatory ON is usually straightforward based on clinical history and the presence of elevated intraocular pressure (IOP) in glaucoma. However, typical features may be absent, ON may have occurred previously, and patients with MS or NMOSD can exhibit subclinical reductions of pRNFL and macular thickness measurements [12]. Moreover, up to half of patients with glaucoma may initially present with IOP measurements within the normal range [13], further complicating the differential diagnosis. In this context, advanced imaging modalities such as OCT and OCT angiography (OCTA) offer the potential to provide objective structural and microvascular biomarkers that may aid clinical evaluation.

Several OCT‐based studies have demonstrated thinning of the pRNFL and macular ganglion cell layer (GCL), as well as alterations in Bruch’s membrane opening minimum rim width (MRW), in glaucoma and other optic nerve disorders, including inflammatory optic neuropathies, and highlighted the value of OCT structural parameters in their differentiation [5, 14, 15]. However, although some differences in pRNFL, GCL, and MRW measurements may be observed between GON and ON eyes, considerable overlap persists, potentially leading to diagnostic uncertainty [5, 15, 16].

Advances in OCT technology have led to the development of OCTA, a dye‐free, noninvasive imaging modality that enables visualization of the retinal, choroid, and optic nerve vessels [17]. OCTA allows the assessment of peripapillary and macular vessel densities (pVD and mVD, respectively), providing additional structural biomarkers for distinguishing glaucoma from other optic neuropathies [4, 18–21]. In GON, reduced pVD and mVD have shown diagnostic and prognostic value and may even precede detectable visual field (VF) loss [22, 23].

OCTA may also be useful for evaluating peripapillary choroidal vessels that are primarily responsible for ONH perfusion [24]. It is believed that dysregulation of blood flow in this region may have a pathogenic role in glaucoma [25]. Previous OCTA‐based studies have identified peripapillary choroidal microvasculature dropout (CMvD) in glaucomatous eyes, reflecting a localized perfusion defect [26, 27]. CMvD has been strongly associated with pRNFL thinning, VF loss, and GON progression [28, 29]. While CMvD was initially proposed as a glaucoma‐specific feature, a few studies have indicated that it may also occur in other optic neuropathies [30, 31].

In inflammatory optic neuropathies, OCTA studies have shown reduced pVD and mVD in eyes with or without prior ON associated with MS and NMOSD, with lower ONH flow index in the choriocapillaris layer, compared to healthy controls [19, 32–34]. However, only two studies have directly compared OCTA findings in NMOSD and glaucoma, reporting significantly lower vessel density in NMOSD than in glaucoma [31, 35]. Accurate differentiation between glaucomatous and inflammatory optic neuropathies is clinically essential, as these conditions differ substantially in prognosis, therapeutics, and long‐term management. Misclassification may potentially result in irreversible visual loss in both GON, MS, and NMOSD, or even severe neurological disabilities in such inflammatory central nervous system diseases. In this context, OCT and OCTA may provide complementary information that improves distinguishing between these entities and contributes to a better understanding of disease‐specific neuroaxonal and vascular alterations. Therefore, the purpose of this study was to compare OCT and OCTA parameters among glaucomatous and inflammatory optic neuropathies.

## 2. Materials and Methods

This prospective cross‐sectional study included eyes of patients with glaucoma (GON group), MS with a history of ON (MS‐ON group), NMOSD with a history of ON (NMOSD‐ON group), and healthy controls (CT group). Participants were recruited from the Neurology and Ophthalmology outpatient clinics at the Federal University of Mato Grosso do Sul. The study adhered to the tenets of the Declaration of Helsinki and was approved by the Institutional Review Board Ethics Committee. Written informed consent was obtained from all participants.

Subjects underwent a comprehensive ophthalmologic examination, including refraction, slit‐lamp biomicroscopy, fundus examination, IOP measurement using Goldmann applanation tonometry, gonioscopy, central corneal thickness (CCT) assessment, and VF testing. A glaucoma and neuro‐ophthalmology specialist performed all examinations. Inclusion criteria were best‐corrected visual acuity (VA) better than 20/200 in the study eye; refractive error within ± 5 diopters for the most ametropic meridian; IOP < 21 mmHg (for controls and patients with MS or NMOSD); absence of ON within the previous 6 months; good cooperation during examinations; and reliable automated perimetry.

All patients with MS and NMOSD had one previous episode of ON, at least 6 months prior to enrollment, and the diagnosis confirmed by clinical, laboratory (including a positive antiaquaporin 4 immunoglobulin G antibodies [AQP4‐IgG] for NMOSD) and imaging criteria. The threshold of 6 months for ON was chosen to minimize the influence of acute and subacute changes, as previous longitudinal studies have shown that the majority of retinal structural alterations, including pRNFL and ganglion cell complex (GCC) thinning, occur within the first 6 months after the acute event [36–38].

The diagnostic criteria for glaucoma required all of the following: (1) characteristic glaucomatous optic disc changes accompanied by abnormal thinning of the pRNFL; (2) VF defects consistent with glaucoma [39], confirmed on at least two separate tests; (3) open anterior chamber angles on gonioscopy; and (4) the absence of ocular or systemic conditions that could cause non‐GON. Only eyes with mild‐stage primary open‐angle glaucoma, as defined by the Hodapp–Anderson–Parrish classification [40], were included in the GON group. Disease severity was determined by the VF mean deviation (MD), with eligibility restricted to eyes with MD better than ‐ 6 dB [18].

The CT eyes were obtained from healthy volunteers with normal ophthalmic and VF findings, recruited from the hospital staff, and comprised two subgroups: 30 eyes from 15 controls who underwent both OCT and OCTA imaging, and 23 eyes from 12 controls who underwent OCT examination only. One control eye was excluded because of corneal opacity.

Exclusion criteria were as follows: age < 18 or > 80 years; media opacities; history of other optic neuropathy or retinal disease; clinical signs of glaucoma (in the MS‐ON and NMOSD‐ON groups); inability to reliably perform VF testing; optic disc anomalies; and a history of alcohol or tobacco abuse, diabetes mellitus, or arterial hypertension requiring treatment with more than three medications.

### 2.1. VF Testing

VF testing was performed using the Humphrey Field Analyzer (Carl Zeiss Meditec, Dublin, CA) with a size III Goldmann stimulus and the Swedish Interactive Threshold Algorithm (SITA) Standard 24‐2 strategy. Reliability criteria were defined as fixation losses ≤ 20%, false‐positive rates ≤ 15%, and false‐negative rates ≤ 30%. VF sensitivity loss was evaluated globally using the MD provided by the equipment.

### 2.2. OCT

Participants underwent OCT and OCTA imaging using a swept‐source device (DRI OCT Triton Plus V.10.11, Topcon, Japan) operating at a wavelength of 1050 nm and an acquisition rate of 100,000 A‐scans per second. Standardized scans covered a 6 × 6 mm area centered on the optic disc and a 7 × 7 mm area of the macula. Only high‐quality images with a signal intensity > 40 and free of motion or blink artifacts were included.

The pRNFL thickness was measured using a circular scan (diameter = 3.4 mm) centered on the ONH. Measurement included global (360°) thickness as well as sectors according to proposed division of the ONH by Garway‐Heath et al. [41]: temporal (T: 310°–41°), superior temporal (ST: 41°–80°), superior nasal (SN: 80°–120°), nasal (N: 121°–230°), inferior nasal (IN: 231°–270°), and inferior temporal (IT: 271°–310°).

The macular retina was automatically segmented by the OCT software, which calculated the combined thickness of the macular retinal nerve fiber layer (mRNFL), GCL, and inner plexiform layer (IPL), collectively referred to as GCC. Macular measurements are derived automatically from the mean thicknesses of the superior and inferior hemiretinas and the global average.

### 2.3. OCTA

OCTA images were acquired using the same device as OCT. The *en face* images were obtained from a 4.5 × 4.5 mm scan centered on the ONH for the peripapillary area and centered on the fovea for the macular area. Images were evaluated for quality and excluded (and image acquisition repeated) if they showed large eye movements (defined as abrupt vessel discontinuities) or black bands due to blinking. Only images with signal quality > 40 were included. OCTA images were obtained and processed using the device’s built‐in software. No specific projection artifact or large vessel removal was performed beyond the default algorithm.

**FIGURE 1:**
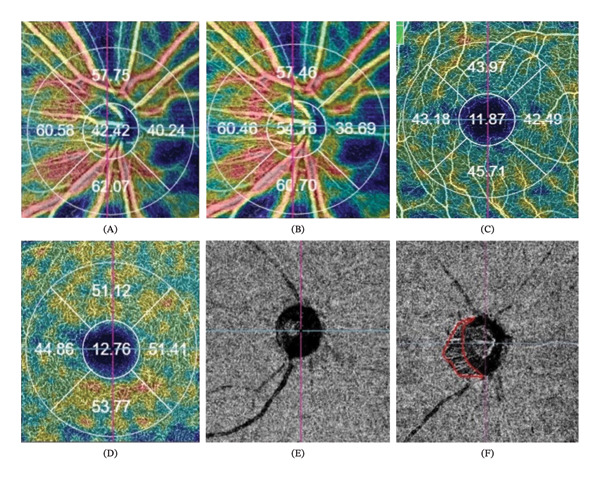
Representative OCTA images showing the superficial optic nerve head vessel density map (A), deep optic nerve head vessel density map (B), superficial macular vessel density map (C), deep macular vessel density map (D), and the peripapillary choroidal layer (E). A focal area of choroid microvascular dropout at the temporal border of the optic disc is shown (F).

Automated segmentation by the IMAGEnet 6 software (Version 1.21.11783) generated *en face* images of distinct vascular layers to obtain the total area of the perfused vasculature per unit area. The superficial pVD (SpVD) was defined as the layer from 2.6 μm below the ILM to 15.6 μm below the interface between the IPL and the inner nuclear layer. Deep peripapillary vessel density (DpVD) was measured from 15.6 μm below the interface between the IPL and the inner nuclear layer to 70.2 μm below this same interface (Figure 1A,B). For the macula, superficial and deep mVD (SmVD and DmVD, respectively) images were obtained using the same segmentation range as the SpVD and DpVD (Figure 1C,D). All images were reviewed to confirm proper centration and segmentation. The vessel densities were expressed in percentage.

Peripapillary choroidal microvasculature was evaluated using *en face* OCTA images of the choroidal layers to assess the presence and area of CMvD (Figure 1E). Images were automatically generated by the OCTA software using a predefined slab extending from the retinal pigment epithelium to 390 μm below it. The CMvD was defined as a focal or sectoral area of absent flow located outside the projected optic disc margin (Figure 1F) in the deep‐layer *en face* image [42] and manually delineated to enable automated area calculation [43]. All scans were carefully reviewed for segmentation accuracy before analysis, and image acquisition was repeated when significant motion or segmentation artifacts were detected. No predefined minimum size threshold for CMvD was adopted, since there is currently no standardized quantitative cutoff established in the literature. Method validation was conducted independently and in a masked fashion by three experienced ophthalmologists using custom validation software that displayed only the original angiographic images. The presence of CMvD was considered when indicated by at least two evaluators and used to obtain the prevalence of such a finding within the groups. Subsequently, the areas with agreement by examiners were calculated using ImageJ software (Version 1.54 g; National Institutes of Health, USA), with metric calibration derived from image metadata. The CMvD area was obtained by averaging the area delimited by the evaluators, and results were expressed in square millimeters (mm^2^).

### 2.4. Data Analysis and Statistics

Descriptive statistics of normally distributed quantitative variables included mean values ± standard deviation (SD). The Shapiro–Wilk test was used to verify the normality of data, and the association between the groups was verified using Student’s *t*‐test. Groups were compared using generalized estimating equation (GEE) to compensate for intereye correlation within the same subject. Continuous outcomes were modeled using an identity link function. Age and sex were included as continuous subject‐level covariates in all GEE models to minimize potential confounding effects related to demographic differences between groups, particularly for OCT and OCTA measurements. An exchangeable working correlation structure was applied, and robust (sandwich) standard errors were used to ensure valid inference. Both eyes were included for all but one subject in the CT and GON groups. In MS‐ON and NMOSD‐ON, the number of eyes varied according to eligibility criteria. The GEE framework appropriately accounts for this unbalanced clustering. Patient‐level characteristics were entered as subject‐level covariates, while OCT and OCTA parameters were analyzed at the eye level within the clustered GEE model. The same modeling approach was consistently applied across all group comparisons.

Interobserver agreement for CMvD analysis was assessed using the intraclass correlation coefficient (ICC) for continuous variables (CMvD area) and Cohen’s kappa coefficient for categorical variables (presence or absence of CMvD). Area under the receiver operating characteristic curve (AUROC) analysis was used to evaluate the ability of OCT and OCTA parameters to differentiate eyes from different disease groups. Correlations between statistically significant OCTA measurements and VA, VF MD, and OCT measurements were verified using Pearson’s correlation test. The presence or absence of CMvD was assessed using Fisher’s exact test. Statistical analyses were performed using IBM SPSS Statistics Version 23.0 (IBM Corp., Armonk, NY, USA), adopting a level of significance of 5% (*p* < 0.05).

## 3. Results

Thirty‐one eyes from 16 patients with glaucoma, 26 eyes from 16 patients with MS, 19 eyes from 12 patients with NMOSD, and 53 eyes from 27 healthy controls were enrolled in the study. The demographic and clinical characteristics of GON, MS‐ON, NMOSD‐ON, and CT groups are summarized in Table 1. VA was significantly higher in the CT and GON groups than in MS‐ON and NMOSD‐ON, with no statistical difference between CT and GON. The NMOSD‐ON group has significantly lower VA than all other groups. Regarding VF MD, values were lowest in the NMOSD‐ON group, followed by the MS‐ON and GON groups, in ascending order.

**TABLE 1:** Clinical and demographic characteristics of glaucoma (GON), multiple sclerosis (MS‐ON), neuromyelitis optica spectrum disorder (NMOSD‐ON), and control (CT) groups.

	Groups				*p* value					
	GON	MS‐ON	NMOSD‐ON	CT	GON × CT	MS‐ON × CT	NMOSD‐ON × CT	GON × MS‐ON	GON × NMOSD‐ON	MS‐ON × NMOSD‐ON
Eyes (patients)	31 (16)	26 (16)	19 (12)	53 (27)	**-**	**-**	**-**	**-**	**-**	**-**
Age (years)	60.80 ± 1.26	32.65 ± 2.10	49.16 ± 1.90	37.94 ± 1.98	**< 0.001**	0.122	**0.006**	**< 0.001**	**< 0.001**	**< 0.001**
Sex (F/M)	10/6	15/1	9/3	16/11	1.000	**0.017**	0.477	0.083	0.687	0.285
VA (decimal)	0.99 ± 0.01	0.80 ± 0.06	0.60 ± 0.08	1.00 ± 0.00	0.301	**< 0.001**	**< 0.001**	**0.008**	**< 0.001**	**0.046**
VF MD (dB)	−3.06 ± 0.35	−5.26 ± 0.77	−11.26 ± 2.38	−0.60 ± 0.16	**< 0.001**	0.242	**< 0.001**	0.960	**0.001**	**0.010**

*Note:* The results are presented as mean ± standard error of the mean. The *p* values were obtained using generalized estimating equations (GEE), except for sex, for which the *p* value was calculated using Fisher’s exact test. Significant values are shown in bold.

Abbreviations: VA = visual acuity, VF MD = visual field mean deviation.

Table 2 shows macular and optic nerve measurements using OCT. The peripapillary and macular parameters were significantly lower in disease groups compared to CT, except for the nasal sector pRNFL in the GON group and the superior nasal sector pRNFL in the MS group. As for the comparison between disease groups, GCC measurements were significantly thinner in both MS‐ON and NMOSD‐ON than in GON, with no significant difference between them. Compared to the GON group, pRNFL measurement was significantly reduced in the temporal sector of MS‐ON and the nasal sector of NMOSD‐ON groups, while pRNFL was significantly reduced in the nasal and superior nasal sector of NMOSD‐ON than MS‐ON groups.

**TABLE 2:** Optical coherence tomography (OCT) measurements of glaucoma (GON), multiple sclerosis (MS‐ON), neuromyelitis optica spectrum disorder (NMOSD‐ON), and control (CT) groups.

OCT parameter	Groups (eyes)				*p* value					
	GON (31)	MS‐ON (26)	NMOSD‐ON (19)	CT (53)	GON × CT	MS‐ON × CT	NMOSD‐ON × CT	GON × MS‐ON	GON × NMOSD‐ON	MS‐ON × NMOSD‐ON
*GCC (μm)*										
Superior	95.22 ± 2.12	83.80 ± 2.39	80.06 ± 4.61	109.32 ± 0.98	**0.001**	**< 0.001**	**< 0.001**	**0.023**	**0.001**	0.594
Inferior	96.14 ± 4.86	85.55 ± 2.66	81.83 ± 4.74	110.04 ± 0.98	**0.009**	**< 0.001**	**< 0.001**	**0.018**	**0.002**	0.518
Average	95.68 ± 2.93	84.85 ± 2.48	80.94 ± 4.65	109.55 ± 0.97	**< 0.001**	**< 0.001**	**< 0.001**	**0.014**	**0.001**	0.585

*pRNFL (μm)*										
Nasal	77.33 ± 2.18	69.71 ± 3.52	57.83 ± 4.18	81.32 ± 1.57	**0.042**	0.063	**< 0.001**	0.080	**0.003**	0.575
Superonasal	96.77 ± 3.97	104.08 ± 5.63	83.36 ± 8.68	114.13 ± 3.47	0.553	0.097	**0.010**	0.584	0.643	0.435
Inferonasal	108.73 ± 4.24	105.96 ± 5.33	92.94 ± 8.63	130.45 ± 2.90	**< 0.001**	**0.002**	**< 0.001**	0.448	0.319	0.573
Temporal	69.57 ± 2.42	53.75 ± 2.99	58.74 ± 5.66	77.38 ± 1.35	**0.011**	**< 0.001**	**< 0.001**	0.177	0.362	0.786
Superotemporal	114.30 ± 4.77	120.71 ± 6.10	100.57 ± 8.43	144.00 ± 2.67	**0.004**	**0.034**	**< 0.001**	0.419	0.399	0.680
Inferotemporal	105.17 ± 6.41	117.38 ± 5.54	108.37 ± 10.11	152.45 ± 3.07	**< 0.001**	**< 0.001**	**< 0.001**	0.447	0.924	0.681
Average	95.31 ± 2.09	95.26 ± 3.89	83.63 ± 6.88	116.62 ± 1.42	**< 0.001**	**< 0.001**	**< 0.001**	0.387	0.322	0.691

*Note:* Mean values (± standard error of mean). A *p* value < 0.05 was considered statistically significant and is shown in bold.

Abbreviations: GCC = ganglion cell complex, pRNFL = peripapillary retinal nerve fiber layer.

As shown in Table 3, only the superior sector of the DmVD was significantly lower in GON compared to CT (52.00% ± 0.58 vs. 54.32% ± 0.61, respectively; *p* value = 0.009). Compared with CT, the NMOSD‐ON group showed significantly lower values in two measurements: the superior sector of the SmVD (46.34% ± 0.92 vs. 49.99% ± 0.64, *p* value = 0.004) and the superior sector of the DmVD (50.95% ± 1.21 vs. 54.32% ± 0.61, respectively; *p* value = 0.023). In addition, the average SmVD was significantly lower in NMOSD‐ON than in GON (45.34% ± 0.81 vs. 47.11% ± 0.33, respectively; *p* value = 0.042). Although several other macular vessel density (mVD) measurements did not reach statistical significance, a consistent trend toward lower mVD values was observed in the NMOSD‐ON group.

**TABLE 3:** Optical coherence tomography angiography (OCTA) measurements of glaucoma (GON), multiple sclerosis (MS‐ON), neuromyelitis optica spectrum disorder (NMOSD‐ON), and control (CT) groups.

OCTA parameter	Groups (eyes)				*p* value					
	GON (31)	MS‐ON (26)	NMOSD‐ON (19)	CT (30)	GON × CT	MS‐ON × CT	NMOSD‐ON × CT	GON × MS‐ON	GON × NMOSD‐ON	MS‐ON × NMOSD‐ON
*Superficial macular vessel density (%)*										
Superior	48.31 ± 0.54	47.38 ± 1.41	46.34 ± 0.92	49.99 ± 0.64	0.150	0.281	**0.005**	0.376	0.061	0.760
Inferior	48.00 ± 0.66	47.81 ± 0.86	46.17 ± 1.16	48.35 ± 1.05	0.767	0.671	**0.018**	0.122	0.124	0.933
Nasal	45.69 ± 0.67	44.05 ± 1.19	43.50 ± 0.97	43.90 ± 0.82	0.246	0.963	0.559	0.735	0.170	0.520
Temporal	46.42 ± 0.49	46.28 ± 0.80	45.36 ± 0.97	46.30 ± 0.57	0.149	0.750	0.663	0.196	0.773	0.615
Average	47.11 ± 0.33	46.38 ± 0.91	45.34 ± 0.81	47.14 ± 0.61	0.746	0.839	0.055	0.340	0.081	0.622

*Deep macular vessel density (%)*										
Superior	52.00 ± 0.58	51.81 ± 1.59	50.95 ± 1.21	54.32 ± 0.61	0.193	0.311	0.051	0.596	0.353	0.716
Inferior	51.49 ± 0.70	51.63 ± 1.10	50.92 ± 1.10	52.48 ± 0.93	0.358	0.895	0.102	0.156	0.342	0.391
Nasal	49.07 ± 0.80	46.63 ± 1.40	47.20 ± 1.19	47.90 ± 0.91	0.375	0.361	0.715	0.576	0.309	0.832
Temporal	49.40 ± 0.55	49.30 ± 1.06	49.31 ± 1.00	49.90 ± 0.63	0.344	0.966	0.990	0.358	0.678	0.997
Average	50.49 ± 0.41	49.84 ± 1.14	49.59 ± 0.93	51.15 ± 0.57	0.958	0.481	0.225	0.406	0.363	0.958

*Superficial vessel density of the optic nerve head (%)*										
Superior	59.46 ± 0.73	62.29 ± 1.36	55.87 ± 2.32	61.72 ± 2.36	0.290	0.677	0.263	0.665	0.172	0.176
Inferior	59.22 ± 1.33	62.75 ± 1.34	57.64 ± 1.67	67.81 ± 0.78	0.058	**0.022**	**< 0.001**	0.953	0.463	0.375
Nasal	51.37 ± 0.96	49.35 ± 1.38	44.80 ± 2.04	50.35 ± 0.98	0.941	0.740	**< 0.001**	**0.037**	**0.039**	0.774
Temporal	49.23 ± 1.05	47.15 ± 0.95	40.76 ± 1.79	52.28 ± 0.73	**0.033**	**< 0.001**	**< 0.001**	0.864	**0.001**	**0.009**
Average	54.82 ± 0.60	55.39 ± 0.84	49.77 ± 1.59	58.04 ± 0.69	**< 0.001**	0.081	**< 0.001**	0.186	**0.006**	0.082

*Deep vessel density of the optic nerve head (%)*										
Superior	58.20 ± 0.72	58.09 ± 2.20	55.38 ± 2.04	62.63 ± 1.04	**< 0.001**	0.258	0.102	0.357	0.183	0.805
Inferior	59.17 ± 1.18	59.70 ± 1.85	58.01 ± 1.60	66.70 ± 0.78	**< 0.001**	0.082	**0.001**	0.594	0.435	0.790
Nasal	50.42 ± 0.98	50.64 ± 1.45	44.81 ± 1.99	50.59 ± 0.98	0.565	0.651	**< 0.001**	0.232	0.078	0.338
Temporal	49.46 ± 1.10	46.94 ± 1.34	40.94 ± 1.70	51.27 ± 0.83	0.539	0.156	**< 0.001**	0.462	**0.003**	0.101
Average	50.02 ± 0.53	50.29 ± 1.44	47.63 ± 1.35	55.28 ± 0.51	**< 0.001**	0.087	**< 0.001**	0.867	0.117	0.079

*CMvD*										
Prevalence (%)	71.0	35.3	26.7	13.3	**< 0.001**	0.136	0.410	**0.031**	**0.010**	0.712
Area (mm^2^)	0.42 ± 0.08	0.07 ± 0.03	0.06 ± 0.04	0.02 ± 0.01	**< 0.001**	0.169	0.271	**0.001**	**0.003**	0.911

*Note:* Mean values (± standard error of mean). A *p* value < 0.05 was considered statistically significant and is shown in bold.

Abbreviation: CMvD = peripapillary choroidal microvasculature dropout.

**FIGURE 2:**
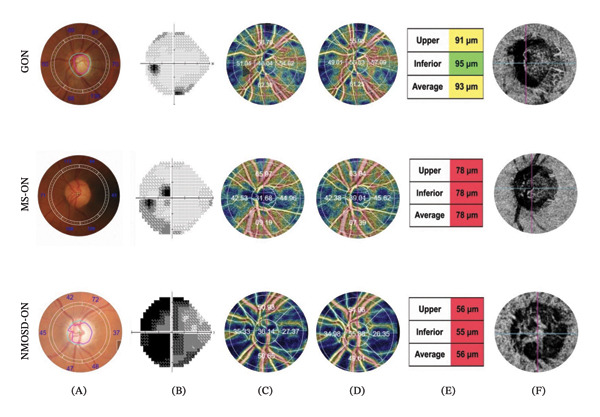
Representative multimodal imaging findings in glaucomatous optic neuropathy (GON), multiple sclerosis (MS‐ON), and neuromyelitis optica spectrum disorder (NMOSD‐ON) groups. Peripapillary retinal nerve fiber layer thickness maps (A), 24‐2 visual field perimetry (B), superficial peripapillary vessel density (C), deep peripapillary vessel density (D), ganglion cell complex thickness (E), and peripapillary choroidal vasculature images (F).

On the other hand, the SpVD analysis revealed several statistically significant differences between disease groups and controls, but also in comparison between GON and NMOSD‐ON groups and NMOSD‐ON *versus* MS‐ON groups. There was no statistical difference between GON and MS regarding SpVD and DpVD measurements. CMvD was observed in all groups, with GON having the highest prevalence and largest area, followed by MS‐ON and NMOSD‐ON (Table 3). The ICC values for CMvD area measurements between examiners 1 vs. 2, 1 vs. 3, and 2 vs. 3 were 0.635 (95% CI: 0.473–0.747), 0.843 (95% CI: 0.773–0.891), and 0.774 (95% CI: 0.673–0.851), respectively, indicating excellent agreement (*p* < 0.001 for all comparisons) among graders for the measurement of CMvD area. Corresponding Cohen’s kappa values were 0.320, 0.405, and 0.453 (*p* < 0.001 for all comparisons), reflecting moderate agreement for the binary classification of CMvD presence. Overall, these results support acceptable reproducibility of CMvD assessment and reinforce the reliability of our methodology. Representative multimodal imaging findings of GON, MS‐ON, and NMOSD‐ON are shown in Figure 2.

ROC curve analysis demonstrated that OCTA‐derived vascular parameters exhibited greater discriminatory ability among GON, MS‐ON, and NMOSD‐ON than isolated structural OCT measurements. The highest AROC values were observed in the superficial and deep temporal sectors of the ONH vessel density, as well as the area of peripapillary CMvD, particularly when comparing glaucoma and NMOSD‐ON (Table 4). Tables 5 and 6, respectively, show the correlation between OCTA parameters and visual function (VA and VF MD) or OCT parameters in all groups of patients. There were several significant correlations in inflammatory optic neuropathy groups, mainly in NMOSD‐ON, but not in the GON group.

**TABLE 4:** Diagnostic performance of OCT and OCTA parameters in differentiating glaucomatous optic neuropathy (GON), multiple sclerosis (MS‐ON), and neuromyelitis optica spectrum disorder (NMOSD‐ON) groups, based on area under the receiver operating characteristic curve (AUROC) analysis.

Variables	GON × MS‐ON		GON × NMOSD‐ON		MS‐ON × NMOSD‐ON	
	*p* value	AUROC (SE)	*p* value	AUROC (SE)	*p* value	AUROC (SE)
*OCT parameters*						
*GCC (μm)*						
Superior	**0.001**	0.75 (0.07)	**0.013**	0.71 (0.09)	0.358	0.58 (0.10)
Inferior	**0.044**	0.66 (0.07)	0.095	0.64 (0.09)	0.383	0.58 (0.10)
Average	**0.011**	0.70 (0.07)	**0.040**	0.67 (0.09)	0.301	0.59 (0.10)

*OCTA parameters*						
Superficial vessel density of the optic nerve head (%)						
Superior	**0.019**	0.68 (0.07)	0.490	0.56 (0.10)	0.053	0.67 (0.08)
Inferior	0.108	0.63 (0.08)	0.478	0.56 (0.09)	**0.039**	0.68 (0.08)
Nasal	0.284	0.42 (0.08)	**0.005**	0.74 (0.08)	0.080	0.66 (0.09)
Temporal	0.152	0.39 (0.08)	**< 0.001**	0.80 (0.07)	**0.008**	0.74 (0.08)
Average	0.434	0.56 (0.08)	**0.023**	0.69 (0.09)	**0.009**	0.73 (0.08)

*Deep vessel density of the optic nerve head (%)*						
Superior	0.227	0.60 (0.09)	0.624	0.54 (0.10)	0.261	0.60 (0.09)
Inferior	0.594	0.54 (0.08)	0.542	0.55 (0.09)	0.284	0.60 (0.09)
Nasal	0.671	0.53 (0.09)	**0.014**	0.71 (0.08)	**0.020**	0.71 (0.08)
Temporal	0.149	0.38 (0.08)	**< 0.001**	0.80 (0.06)	**0.013**	0.73 (0.08)
Average	0.311	0.58 (0.09)	0.212	0.61 (0.10)	0.090	0.65 (0.09)

*CMvD*						
Area (mm^2^)	0.097	0.63 (0.08)	**0.001**	0.78 (0.07)	**0.046**	0.68 (0.09)

*Note:* Statistically significant values are shown in bold.

Abbreviations: CMvD = choroidal microvascular dropout, GCC = ganglion cell complex, OCT = optical coherence tomography, OCTA = optical coherence tomography angiography, SE = standard error.

**TABLE 5:** Pearson’s correlation coefficients (*r*) between the OCTA and OCT parameters in glaucomatous optic neuropathy (GON), multiple sclerosis (MS‐ON), and neuromyelitis optica spectrum disorder (NMOSD‐ON) groups.

OCTA parameter	MS‐ON		NMOSD‐ON		GON	
	pRNFL	GCC	pRNFL	GCC	pRNFL	GCC
SmVD	*r* = 0.653	*r* = 0.415	*r* = 0.666	*r* = 0.680	*r* = −0.129	*r* = 0.063
	***p* = 0.001**	***p* = 0.035**	***p* = 0.002**	***p* = 0.001**	*p* = 0.497	*p* = 0.736

DmVD	*r* = 0.549	*r* = 0.458	*r* = 0.472	*r* = 0.564	*r* = −0.270	*r* = −0.086
	***p* = 0.008**	***p* = 0.024**	***p* = 0.041**	***p* = 0.012**	*p* = 0.149	*p* = 0.644

SpVD	*r* = 0.773	*r* = 0.645	*r* = 0.935	*r* = 0.877	*r* = 0.257	*r* = 0.025
	***p* < 0.001**	***p* = 0.001**	***p* < 0.001**	***p* < 0.001**	*p* = 0.171	*p* = 0.849

DpVD	*r* = 0.294	*r* = 0.405	*r* = 0.927	*r* = 0.847	*r* = 0.220	*r* = 0.187
	*p* = 0.173	***p* = 0.045**	***p* < 0.001**	***p* < 0.001**	*p* = 0.242	*p* = 0.314

CMvD—area	*r* = 0.389	*r* = 0.294	*r* = 0.771	*r* = 0.775	*r* = 0.276	*r* = 0.205
	*p* = 0.060	*p* = 0.146	***p* < 0.001**	***p* < 0.001**	*p* = 0.147	*p* = 0.278

*Note:* A *p* value < 0.05 was considered statistically significant and is shown in bold.

Abbreviations: DmVD = deep macular vessel density, DpVD = deep peripapillary vessel density, GCC = ganglion cell complex, pRNFL = peripapillary retinal nerve fiber layer, SmVD = superficial macular vessel density, SpVD = superficial peripapillary vessel density.

**TABLE 6:** Pearson’s correlation coefficients (*r*) between the OCTA parameters, visual acuity (VA), and visual field mean deviation (VF MD) in glaucomatous optic neuropathy (GON), multiple sclerosis (MS‐ON), and neuromyelitis optica spectrum disorder (NMOSD‐ON) groups.

OCTA parameter	MS‐ON		NMOSD‐ON		GON	
	VA	VF MD	VA	VF MD	VA	VF MD
SmVD	*r* = 0.488	*r* = 0.745	*r* = 0.523	*r* = 0.649	*r* = 0.165	*r* = 0.159
	***p* = 0.011**	***p* < 0.001**	***p* = 0.022**	***p* = 0.004**	*p* = 0.374	*p* = 0.402

DmVD	*r* = 0.577	*r* = 0.737	*r* = 0.472	*r* = 0.519	*r* = 0.163	*r* = −0.019
	***p* = 0.003**	***p* < 0.001**	***p* = 0.042**	***p* = 0.027**	*p* = 0.380	*p* = 0.919

SpVD	*r* = 0.340	*r* = 0.366	*r* = 0.571	*r* = 0.818	*r* = 0.048	*r* = 0.106
	*p* = 0.097	*p* = 0.072	***p* = 0.011**	***p* < 0.001**	*p* = 0.798	*p* = 0.576

DpVD	*r* = 0.021	*r* = 0.503	*r* = 0.490	*r* = 0.711	*r* = 0.220	*r* = 0.278
	*p* = 0.922	***p* = 0.010**	***p* = 0.033**	***p* = 0.001**	*p* = 0.235	*p* = 0.137

CMvD – area	*r* = 0.063	*r* = −0.233	*r* = 0.013	*r* = 0.251	*r* = 0.005	*r* = −0.120
	*p* = 0.787	*p* = 0.309	*p* = 0.963	*p* = 0.386	*p* = 0.981	*p* = 0.529

*Note:* A *p* value < 0.05 was considered statistically significant and is shown in bold.

Abbreviations: DmVD = deep macular vessel density, DpVD = deep peripapillary vessel density, SmVD = superficial macular vessel density, SpVD = superficial peripapillary vessel density.

## 4. Discussion

It is well known that VA and VF are affected by ON, typically with more severe damage in NMOSD [7]. In contrast, VA is often preserved in mild‐to‐moderate GON, although VF MD worsens with disease progression [40]. Since this study enrolled only patients with mild GON and, in the inflammatory optic neuropathy groups, patients with a history of ON, the observed differences in VA and VF MD between GON, MS‐ON, NMOSD‐ON, and CT were expected and are consistent with previous reports [18, 45]. Although patients with MS‐ON and NMOSD‐ON had worse VA than those with GON, the similar VF MD between GON and MS‐ON, together with the more severe VF sensitivity loss in the NMOSD‐ON group, suggests a less severe ON in MS than NMOSD [46]. These findings are also in agreement with the expected greater central involvement in MS and NMOSD compared with GON, whereas GON tends to cause relatively more peripheral visual damage than the two other diseases [9].

Despite the differences in VA and VF MD among groups, OCT measurements identified only one pRNFL sector differentiating GON from NMOSD‐ON (the nasal sector). Other OCT‐based studies have shown that Bruch’s membrane opening MRW, as well as the MRW‐to‐pRNFL ratio, may be more effective than pRNFL alone in differentiating GON from other non‐GONs [5, 14].

Our findings reinforce the notion that pRNFL thickness alone may not be sufficient to differentiate GON from MS‐ON or NMOSD‐ON. Consistent with the findings reported by Rogaczewska et al. in MS, the superonasal and nasal pRNFL sectors did not show statistically significant changes when compared to CT in our cohort, suggesting that this region may be the least affected by the disease [47]. When comparing NMOSD‐ON with MS‐ON or GON, the nasal pRNFL sector was more markedly affected than other sectors. This finding suggests more diffuse and severe optic nerve damage in NMOSD following ON, as previously described in the literature [48]. Thus, in a clinical setting with suspected NMOSD, greater thinning of the nasal sectors after an ON attack may help differentiate NMOSD from GON and MS.

In turn, the GCC was thinner in the MS‐ON and NMOSD‐ON than in the GON group, with no significant difference between MS‐ON and NMOSD‐ON. These findings may reflect the relative preservation of the macular region in the early stages of GON, despite substantial pRNFL loss and optic disc cupping typically observed in glaucoma, as well as the more diffuse involvement of both pRNFL and GCC following ON in MS and NMOSD [49, 50]. Overall, our results suggest that GCC thickness may aid in distinguishing GON from MS‐ON and NMOSD‐ON, but less so for differentiating between the two inflammatory optic neuropathies.

Our study also revealed that OCTA measurements, particularly at the level of the ONH, may help differentiate glaucoma, MS‐ON, and NMOSD‐ON. In contrast to structural OCT findings, the mVD appeared to be less markedly affected across disease groups, although a trend toward lower values was observed in the NMOSD‐ON group without reaching statistical significance in most sectors (Table 3). However, given the limited sample size, particularly in the NMOSD‐ON group, these findings should be interpreted with caution, and subtle macular vascular alterations cannot be excluded. In turn, the pVD was markedly reduced. Both superficial and deep capillary plexuses showed lower densities in several parameters in GON, MS‐ON, and NMOSD‐ON compared to CT, indicating greater vascular involvement in the optic nerve.

Our OCTA findings in MS are consistent with those reported by Wang et al., who observed decreased blood flow in the ONH, but did not identify significant changes in the macular region [20]. The reduction in pVD may be explained by axonal damage and the consequent loss of optic nerve fibers following ON. The resulting decrease in metabolic demand may lead to secondary reductions in blood flow through autoregulatory vascular mechanisms. Another hypothesis involves endothelial abnormalities, previously documented in patients with MS, which may impair microvascular function and further contribute to the diminished perfusion at the ONH [20, 51]. The preserved mVD in MS should be explained by the preferential damage of the innermost retinal layers, typically sparing the middle and deep retinal layers. As a result, these preserved layers may retain sufficient functional integrity to maintain effective autoregulation of macular blood flow [20]. Previous study supports the hypothesis that NMOSD may present a retinal vasculopathy driven by AQP4‐related astroglial dysfunction, in addition to damage to ocular tissues caused by ON [52]. It is important to note that in our study, the nasal sectors in both SpVD and DpVD were significantly lower in NMOSD‐ON than GON, MS‐ON, and CT groups, while GON and MS‐ON did not differ statistically from controls in such sectors. Therefore, the vessels of the nasal sector of the ONH seem to be more affected by NMOSD than GON or MS and, on average, NMOSD‐ON presents lower pVD compared to MS‐ON, with both findings helping in their differentiation. The combination of OCT and OCTA findings also suggests a pattern of diffuse and more severe damage of the pRNFL, GCC, and ONH vessels in NMOSD and reinforces the substantial neuronal loss and retinal vasculopathy associated with NMOSD‐related optic neuropathy [53, 54].

CMvD was previously thought to occur almost exclusively in GON [54, 55] largely because it is typically located within the margin of β‐parapapillary atrophy (βPPA). However, more recent studies revealed that CMvD may also be present in other optic neuropathies [30, 43]. To our knowledge, this is the first study to compare the CMvD among GON, MS‐ON, and NMOSD‐ON. Our results corroborate previous reports, demonstrating a high prevalence of CMvD in GON and suggesting an association with this condition, although this finding should not be considered disease‐specific since it was also identified in inflammatory optic neuropathies (MS and NMOSD) and in healthy controls. The presence of CMvD in a small number of healthy controls, as shown in this study, has also been previously demonstrated in the literature, reinforcing our findings that CMvD is not exclusive of GON [44]. In addition to being more prevalent, CMvD had a larger area in GON than in MS‐ON and NMOSD‐ON, despite the inclusion of only mild‐stage glaucoma cases. The MS‐ON and NMOSD‐ON groups did not differ significantly in either CMvD prevalence or area.

Compared with NMOSD‐ON, GON showed both higher CMvD prevalence and a larger CMvD area, even though NMOSD‐ON exhibited lower pVD. GON also presented larger CMvD area than MS‐ON, although no differences were found between these two groups in CMvD prevalence. Taken together, these results suggest more pronounced damage to deep ONH vessels in GON and NMOSD, with relatively less involvement in MS. In addition, peripapillary choroidal vessels appear to be more severely affected in GON than in the inflammatory optic neuropathies. It is important to note that the moderate inter‐rater agreement values indicate that CMvD assessment remains partially subjective and may limit reproducibility and clinical applicability, highlighting the need for more objective quantitative characterization of choroidal microvasculature in future studies.

In the AUROC analysis, OCTA‐derived vascular parameters demonstrated better discriminatory performance among disease groups than structural OCT measurements alone. While GCC thickness showed moderate ability to differentiate GON from inflammatory optic neuropathies, superficial and DpVD measurements, particularly in the temporal and nasal sectors, achieved the highest AUROC values. In addition, the CMvD area showed good discriminatory ability, especially between GON and NMOSD‐ON. These findings suggest that different optic neuropathies exhibit distinct patterns of microvascular impairment and reinforce the potential role of OCTA as a complementary tool for the differential diagnosis of glaucomatous and inflammatory optic neuropathies.

The evaluation of structure–function is important in many optic neuroophthalmological disorders, adding to diagnosis, prognosis, and patient follow‐up [56–58]. However, in some cases, such as in early‐stage glaucoma, the correlation between structural exams and functional data may be weak [59]. We have found no correlation between the OCTA parameters and VA or VF MD or OCT parameters in the GON group (Tables 5 and 6). However, this finding should be interpreted with caution, since only eyes with mild glaucoma were included, which may have limited the ability to detect structure‐function correlations. Furthermore, the CMvD does not seem to be associated with VA or VF loss in early‐stage glaucoma.

Regarding the inflammatory optic neuropathies, the MS‐ON group showed a moderate to strong structure–function correlation between superficial and deep mVD and VA and VF MD, with fewer associations with the ONH vasculature and no association with CMvD. This finding reinforces the importance of monitoring not only the retinal GCL but also possible macular and ONH vascular changes in patients with MS, regardless of the history of ON [60]. On the other hand, NMOSD‐ON showed moderate‐to‐strong correlations between visual functional data (VA and VF MD) and all structural OCTA parameters, except for CMvD. In addition, these correlations suggest a probable relationship between superficial and deep macular and ONH vessels in NMOSD pathogenesis and emphasize the hypothesis of a retinal vasculopathy associated with the disease [61].

A significant correlation was observed between OCTA parameters and OCT‐measured pRNFL and GCC in MS‐ON and NMOSD‐ON patients. These findings indicate that, in inflammatory optic neuropathies, particularly NMOSD, microvascular impairment is closely associated with structural neural loss, supporting a potentially integrated pathophysiological mechanism linking inflammation, vascular dysfunction, and axonal degeneration. In contrast, the absence of significant correlations in mild glaucoma suggests that structural damage may occur independently of OCTA‐detectable vascular alterations or that the temporal relationship between vascular compromise and neural damage differs in this condition.

This study has some limitations that should be acknowledged. First, its cross‐sectional design precludes the establishment of causal relationships and does not allow evaluation of disease progression. Therefore, the observed structural and vascular differences should be interpreted as associations rather than evidence of temporal or causal effects. Nevertheless, the cross‐sectional approach remains appropriate for identifying differences between groups and provides relevant insights into disease‐related patterns of structural and microvascular alterations. Another limitation of this study is the variability in the time elapsed since ON among patients with MS and NMOSD. The use of a 6‐month threshold likely captures the chronic phase of axonal loss while reducing variability related to acute or subacute processes. Nonetheless, residual variability related to disease duration cannot be completely excluded and should be considered when interpreting the findings. The relatively small sample size, particularly in the NMOSD‐ON group, may limit statistical power and reduce the generalizability of the findings. Also, since this was an exploratory study, *p* values were not corrected for multiple comparisons, and some significant results may in fact be false‐positive. Although image quality was carefully reviewed and standardized acquisition protocols were applied across all groups, potential influence of segmentation errors and imaging artifacts inherent to OCTA imaging may still have introduced variability in the results. Furthermore, given that more advanced neural damage is expected to result in greater structural and vascular changes, some of the observed differences between groups may reflect variations in disease severity rather than disease‐specific mechanisms. In addition, significant age differences between groups may have influenced OCT and OCTA measurements, particularly vascular parameters known to be age sensitive. Although age was included as a continuous covariate in all GEE models, residual age‐related confounding cannot be completely excluded. Furthermore, the inclusion of only mild‐stage glaucoma cases may have reduced the ability to detect structure–function correlations in the GON group and limits extrapolation of these findings to more advanced stages of the disease. Therefore, these findings should be interpreted with caution, particularly at the clinical and individual patient level when considering their potential diagnostic implications.

In conclusion, our findings suggest that the superficial and deep ONH vessels are more affected in NMOSD than GON or MS, but CMvD is more prevalent and larger in GON. Also, MS seems to have a predilection for involvement of the temporal and inferior sectors in both plexuses of the ONH compared to healthy controls, although OCTA changes seem to be less distinct than in GON and NMOSD. In NMOSD, in turn, we have found a pattern of greater involvement of the ONH plexuses in a setting of relatively preserved choroidal vessels. These different patterns of OCTA abnormalities may aid in their differentiation in the clinical setting. Furthermore, further longitudinal studies are needed to evaluate whether these OCTA biomarkers may be useful in evaluating disease progression, prognosis, and patient management.

## Funding

This study was supported by grants from Coordenação de Aperfeiçoamento do Pessoal de Nível Superior (Grant no 4951‐10‐07), Brasília, Brazil, and CNPq—Conselho Nacional de Desenvolvimento Científico e Tecnológico (no. 311811/2022‐1), Brasília, Brazil.

## Disclosure

The funding organizations had no role in the design or conduct of this research.

## Conflicts of Interest

The authors declare no conflicts of interest.

## Data Availability

The data that support the findings of this study are available from the corresponding author upon reasonable request.
